# Helminth defence molecules—immunomodulators designed by parasites!

**DOI:** 10.3389/fmicb.2013.00296

**Published:** 2013-10-01

**Authors:** Mark W. Robinson, Sheila Donnelly, John P. Dalton

**Affiliations:** ^1^Medical Biology Centre, School of Biological Sciences, Queen's University BelfastBelfast, Northern Ireland; ^2^The ithree Institute, University of Technology SydneyUltimo, Sydney, NSW, Australia; ^3^Institute of Parasitology, McGill UniversitySt-Anne-de-Bellevue, QC, Canada

**Keywords:** parasite, helminth, *Fasciola*, *Schistosoma*, trematodes, immunomodulator, helminth defence molecule, antimicrobial peptide

## Helminth defence molecules (HDMs)—antimicrobials or immunomodulators?

Parasitic helminths (worms) are one of the most successful animal groups in nature. They are large multicellular organisms and therefore cannot penetrate host cells but must reside inside tissue or organs. They infect over 1 billion people globally, mostly in tropical/sub-tropical regions, taking an enormous toll on animal and human health (Hotez et al., 2008). Although the main evolutionary driving force for parasitism may have been the ease of access to food this brought other challenges, most importantly the need to overcome expulsion by the immune responses of the host. Accordingly, helminths have evolved elaborate mechanisms to manage, suppress or manipulate the mammalian immune system.

It is generally thought that worms influence the host immune response by secreting factors into their environment—the host parasite interface. Over the past 10 years the application of proteomics techniques has allowed us to identify molecules secreted by helminths. Although the exact complement of secretory molecules differs between species, most helminths release proteolytic enzymes with endo- and exo-peptidase activities (Robinson et al., 2008a,b), antioxidants such as glutathione-*S* transferase and peroxiredoxin (Jefferies et al., 2001; Donnelly et al., 2008; Robinson et al., 2009) and other molecules with a range of biochemical activities including protease inhibitors and metabolic enzymes. Whilst the biological function of a few of these helminth molecules are well defined, this is generally restricted to those that possess evolutionarily conserved catalytic domains/active site residues that are easily identified using bioinformatics search tools (e.g., *Fasciola* cysteine proteases function in fluke nutrition; Lowther et al., 2009). However, many helminths also secrete a range of molecules whose primary sequences offer no clue as to their biochemical activity or biological function.

During our on-going analysis of the secretory proteome (i.e., the secretome) of the helminth *Fasciola hepatica*, a parasite that infects its host via the intestine following ingestion and then migrates to the liver, we identified a novel and abundant 8 kDa protein. The protein contained a secretory signal peptide but BLAST analysis of its primary sequence failed to infer a function. However, structural studies revealed that a 37 amino acid C-terminal region adopted a similar secondary structure (amphipathic α-helix) to a number of peptides with known antimicrobial and/or immunomodulatory functions, most particularly mammalian LL-37 (Robinson et al., 2011). Phylogenetic analysis discovered that these secretory proteins are conserved across the major trematode species that collectively infect >1 billion humans, including the liver flukes *Clonorchis sinensis*, *Paragonimus westermani*, and *Opisthorchis viverrini* and the blood flukes *Schistosoma* *mansoni* and *S. japonicum* (Robinson et al., 2011). Accordingly, by analogy with the mammalian antimicrobial and/or immunomodulators we hypothesized that these helminth molecules may play a critical role in the parasites interaction with its host and thus named them helminth defence molecules (HDMs). This hypothesis, however, posed the question that is central to understanding how they may perform this function, namely, are HDMs antimicrobial or immunomodulatory peptides, or both?

## HDMs do not possess antimicrobial or haemolytic activity

To begin to address this question, we compared the ability of helminth-derived HDMs with several well-known mammalian host-derived antimicrobial peptides [AMPs; also termed host defence peptides (HDPs) where they display immunomodulatory activities] to directly kill clinically-relevant microorganisms (Thivierge et al., 2013). The antimicrobial effect of AMPs/HDPs is attributed to the ability of their amphipathic helices to bind, and thus disrupt, negatively charged bacterial cell membranes (Hancock and Chapple, 1999). Despite having similar biochemical/biophysical properties to the host-derived AMPs/HDPs including LL-37, CRAMP, SMAP-29, and BMAP-28 which variously showed activity against both gram-negative (*Escherichia coli*, *Pseudomonas aeruginosa*, and *Salmonella typhimurium*) and gram-positive (*Staphylococcus epidermis* and *Staphylococcus aureus*) bacteria, none of the HDMs tested demonstrated bactericidal activity against any species of bacteria at the concentrations tested (<0.25–128 μg/ml; Thivierge et al., 2013).

Previous studies had also shown that AMPs/HDPs are cytotoxic to kinetoplastid protozoan parasites such as *Leishmania major* (Lynn et al., 2011), *Trypanosoma cruzi* (Haines et al., 2009), and the apicomplexan protozoan parasites *Cryptosporidium* sp. (Carryn et al., 2012). In our studies, whilst we confirmed that several vertebrate AMPs/HDPs killed the apicomplexan protozoan parasite *Cryptosporidium parvum* and *C. hominis in vitro* at concentrations as low as 0.025 μM none of the HDMs tested displayed any parasiticidal activity even at 2.5 μM (Thivierge et al., 2013).

The predominant mechanism of AMP/HDP bactericidal activity is the formation of pores in the membrane lipid bilayer, destroying its integrity and causing cell death (Oren and Shai, 1998). However, this effect is not specific to bacterial cells since eukaryotic cells can also be lysed via this mechanism (Ciornei et al., 2005). Whilst the mammalian AMPs/HDPs rapidly lysed red blood cells in a concentration-dependent manner (from 8 to 256 μg/ml), the HDMs did not induce significant lysis at equivalent concentrations (Thivierge et al., 2013). Using a fluorescent membrane-impermeant dye we demonstrated that the mammalian peptides LL-37, CRAMP, SMAP-29, and BMAP-28 (50 μM) induced the formation of pores in a murine macrophage cell line and in general, were cytotoxic at concentrations of >25 μM. However, at the same concentrations none of the helminth peptides exhibited these lytic or cytotoxic effects (Thivierge et al., 2013).

The contrasting effects of the mammalian AMPs/HDPs and the helminth HDMs may be due to the targeting of specific membrane components by HDMs necessary for internalization into host cells rather than non-specific “carpet” binding to the phospholipid bilayer that may occur with the AMPs/HDPs (Brender et al., 2012). We have shown that *F. hepatica* HDM (FhHDM-1) binds to host macrophage plasma membrane lipid rafts, possibly via selective interaction with cholesterol, before being internalized by endocytosis (Robinson et al., 2012). In contrast, human LL-37 cannot bind to cholesterol; indeed its presence strongly reduces the ability of LL-37 to interact with phospholipid membranes (Sood and Kinnunen, 2008). Whilst the precise mechanism(s) governing the selectivity of the HDMs vs. AMPs/HDPs is not fully understood, our observations show that, rather than simply destroying host cells by lysis, HDMs have evolved specifically to interact with host cell membranes without causing their disruption.

## HDMs display a variety of immunomodulatory activities

Parasitic helminths secrete a range of soluble effector molecules that modulate host immune responses in a myriad of ways to establish an environment that facilitates their survival and a prolonged reproductive phase (reviewed by Harnett and Harnett, 2010). We have previously shown that *F. hepatica* and *S. mansoni* secrete molecules with specific immunomodulatory functions: peroxiredoxin (Prx) promotes the development of host Th2 responses via the induction of M2 macrophages (Donnelly et al., 2005, 2008) and cathepsin L1 (FhCL1) inhibits the macrophage MyD88-independant, TRIF-dependant signaling pathway via cleavage of toll-like receptor (TLR) 3 within the endosome (Donnelly et al., 2010). However, our recent studies have shown that HDMs are “utility players” in the host-parasite interaction and exert multiple effects on host immune cells.

In order to infect their mammalian host, the infective stage of *F. hepatica*, termed newly excysted juveniles (NEJs), secrete an array of cysteine peptidases including cathepsins B and cathepsins L that digest a path through the intestinal wall (McGonigle et al., 2008; Robinson et al., 2009). Despite this loss of barrier function of the intestinal epithelium and consequent translocation of luminal antigens (bacteria and their toxins) into the circulation, potent host responses such as septicaemia are not common events during helminth infections. We found that FhHDM-1 binds directly to *E. coli* lipopolysaccharide (LPS) preventing its interaction with the TLR4/MD2/CD14 complex on the macrophage surface (Robinson et al., 2011). FhHDM-1 also exhibited a striking ability to protect mice against LPS-induced inflammation by preventing the release of inflammatory mediators (TNF and IL-1β) from macrophages (Robinson et al., 2011).

IFNγ is one of the key cytokines in the innate immune response to intracellular pathogens, and augments cellular responses to TLR ligands such as bacterial LPS (Held et al., 1999; Schroder et al., 2006). We also found that, like the mammalian peptides LL-37, CRAMP, SMAP-29, and BMAP-28, HDMs significantly inhibited macrophage TNF production in response to combined stimulation with LPS and IFNγ (Thivierge et al., 2013). Both AMPs/HDPs and HDMs can seemingly also inhibit inflammatory macrophages using mechanisms that are independent of direct binding to LPS (Brown et al., 2011; Thivierge et al., 2013). Thus, the secretion of HDMs by the parasite may protect the host against excessive bacterial-induced inflammation that would otherwise occur during migration of the parasite through the host intestinal wall due to concurrent translocation of luminal bacteria. By offering this protection the parasite enhances the survival of its host and, accordingly, its own longevity.

Confocal microscopy, using fluorescently labeled peptides, has shown that after initial interaction with lipid rafts on the macrophage surface, FhHDM-1 enters the cell via the endolysosomal pathway (Robinson et al., 2012). FhHDM-1 is cleaved by endogenous (host) cathepsin L to specifically release a C-terminal peptide (containing the conserved HDM amphipathic helix) which then prevents the acidification of the endolysosomal compartments by inhibiting vacuolar (v) ATPase activity. Uncoupling endolysosomal acidification impedes macrophage antigen processing by proteases, such as cathepsin L, preventing the presentation of peptides at the cell surface in conjunction with MHC class II to CD4 + T cells (Robinson et al., 2012). By suppressing the antigen presenting function of host macrophages, HDM indirectly impairs the subsequent development of adaptive immune responses against the parasite. However, by altering the secretion of immunoglobulins from activated B cells, HDMs are also capable of directly influencing the host adaptive response. HDMs enhanced the IL-4 induced production of IgG1 and suppressed the release of IgG2a from murine B cells in response to IFNγ (Thivierge et al., 2013). This is consistent with a wound healing scenario (Nishio et al., 2009) which, again, may serve to protect the host from helminth-induced tissue damage.

## Parasite-designed HDMs may have therapeutic potential

The immune-modulatory properties of mammalian HDPs, and in particular their ability to prevent excessive immunopathology associated with bacterial sepsis, has attracted interest in exploiting these as anti-infectives and immunotherapeutic agents (Easton et al., 2009). However, their clinical development has been hampered by the occurrence of toxic off-target effects and cell lysis. Efforts to improve delivery of HDPs to their desired site of action (e.g., by conjugation with targeting moieties) are on-going with the aim of enhancing efficacy whilst reducing deleterious side effects (Devocelle, 2012). However, helminth-derived HDMs may represent a more attractive therapeutic option: they show all the potent immunomodulatory effects of the HDPs without the cytotoxic and cytolytic effects (Thivierge et al., 2013). We are currently determining the translatability of the immune-modulatory effect of HDMs from murine to human cells rather than screening an array of animal models of disease (Robinson et al., 2013) and we are hopeful that the therapeutic potential of these parasite-designed molecules can be realised (Donnelly et al., 2011).

## References

[B1] Brender J. R. McHenry A. J. Ramamoorthy A. (2012). Does cholesterol play a role in the bacterial selectivity of antimicrobial peptides? Front. Immunol. 3:195 10.3389/fimmu.2012.00195 22822405 PMC3398343

[B2] Brown K. L. Poon G. F. Birkenhead D. Pena O. M. Falsafi R. Dahlgren C. (2011). Host defense peptide LL-37 selectively reduces proinflammatory macrophage responses. J. Immunol. 186, 5497–5505 10.4049/jimmunol.1002508 21441450

[B3] Carryn S. Schaefer D. A. Imboden M. Homan E. J. Bremel R. D. Riggs M. W. (2012). Phospholipases and cationic peptides inhibit *Cryptosporidium parvum* sporozoiteinfectivity by parasiticidal and non-parasiticidal mechanisms. J. Parasitol. 98, 199–204 10.1645/GE-2822.1 21787211

[B4] Ciornei C. D. Sigurdardottir T. Schmidtchen A. Bodelsson M. (2005). Antimicrobial and chemoattractant activity, lipopolysaccharide neutralization, cytotoxicity, and inhibition by serum of analogs of human cathelicidin LL-37. Antimicrob. Agents Chemother. 49, 2845–2850 10.1128/AAC.49.7.2845-2850.2005 15980359 PMC1168709

[B5] Devocelle M. (2012). Targeted antimicrobial peptides. Front. Immunol. 3:309 10.3389/fimmu.2012.00309 23060887 PMC3464401

[B6] Donnelly S. O'Neill S. M. Sekiya M. Mulcahy G. Dalton J. P. (2005). Thioredoxin peroxidase secreted by *Fasciola hepatica* induces the alternative activation of macrophages. Infect. Immun. 73, 166–173 10.1128/IAI.73.1.166-173.2005 15618151 PMC538930

[B7] Donnelly S. O'Neill S. M. Stack C. M. Robinson M. W. Turnbull L. Whitchurch C. (2010). Helminth cysteine proteases inhibit TRIF–dependent activation of macrophages via degradation of TLR3. J. Biol. Chem. 285, 3383–3392 10.1074/jbc.m109.060368 19923225 PMC2823461

[B8] Donnelly S. Robinson M. W. Dalton J. P. To J. (2011). Immune modulating agents and uses therefor. PCT/AU2011/001402.

[B9] Donnelly S. Stack C. M. O'Neill S. M. Sayed A. A. Williams D. L. Dalton J. P. (2008). Helminth 2-Cys peroxiredoxin drives Th2 responses through a mechanism involving alternatively activated macrophages. FASEB J. 22, 4022–4032 10.1096/fj.08-106278 18708590 PMC3980656

[B10] Easton D. M. Nijnik A. Mayer M. L. Hancock R. E. (2009). Potential of immunomodulatory host defense peptides as novel anti-infectives. Trends Biotechnol. 27, 582–590 10.1016/j.tibtech.2009.07.004 19683819 PMC7114281

[B11] Haines L. R. Thomas J. M. Jackson A. M. Eyford B. A. Razavi M. Watson C. N. (2009). Killing of trypanosomatid parasites by a modified bovine host defense peptide, BMAP-18. PLoS Negl. Trop. Dis. 3:e373 10.1371/journal.pntd.0000373 19190729 PMC2628741

[B12] Hancock R. E. Chapple D. S. (1999). Peptide antibiotics. Antimicrob. Agents Chemother. 43, 1317–1323 10348745 10.1128/aac.43.6.1317 PMC89271

[B13] Harnett W. Harnett M. M. (2010). Helminth-derived immunomodulators: can understanding the worm produce the pill? Nat. Rev. Immunol. 10, 278–284 10.1038/nri2730 20224568

[B14] Held T. K. Weihua X. Yuan L. Kalvakolanu D. V. Cross A. S. (1999). Gamma interferon augments macrophage activation by lipopolysaccharide by two distinct mechanisms, at the signal transduction level and via an autocrine mechanism involving tumor necrosis factor alpha and interleukin-1. Infect. Immun. 67, 206–212 9864217 10.1128/iai.67.1.206-212.1999 PMC96298

[B15] Hotez P. J. Brindley P. J. Bethony J. M. King C. H. Pearce E. J. Jacobson J. (2008). Helminth infections: the great neglected tropical diseases. J. Clin. Invest. 118, 1311–1321 10.1172/JCI34261 18382743 PMC2276811

[B16] Jefferies J. R. Campbell A. M. van Rossum A. J. Barrett J. Brophy P. M. (2001). Proteomic analysis of *Fasciola hepatica* excretory-secretory products. Proteomics 1, 1128–1132 11990507 10.1002/1615-9861(200109)1:9<1128::AID-PROT1128>3.0.CO;2-0

[B17] Lowther J. Robinson M. W. Donnelly S. M. Xu W. Stack C. M. Matthews J. M. (2009). The importance of pH in regulating the function of *Fasciola hepatica* cathepsin L1 cysteine protease. PLoS Negl. Trop. Dis. 3:e369 10.1371/journal.pntd.0000369 19172172 PMC2621350

[B18] Lynn M. A. Kindrachuk J. Marr A. K. Jenssen H. Pante N. Elliott M. R. (2011). Effect of BMAP-28 antimicrobial peptides on Leishmania major promastigote and amastigote growth: role of leishmanolysin in parasite survival. PLoS Negl. Trop. Dis. 5:e1141 10.1371/journal.pntd.0001141 21655347 PMC3104953

[B19] McGonigle L. Mousley A. Marks N. J. Brennan G. P. Dalton J. P. Spithill T. W. (2008). The silencing of cysteine proteases in *Fasciola hepatica* newly excysted juveniles using RNA interference reduces gut penetration. Int. J. Parasitol. 38, 149–155 10.1016/j.ijpara.2007.10.007 18048044

[B20] Nishio N. Ito S. Suzuki H. Isobe K. (2009). Antibodies to wounded tissue enhance cutaneous wound healing. Immunology 128, 369–380 10.1111/j.1365-2567.2009.03119.x 20067537 PMC2770685

[B21] Oren Z. Shai Y. (1998). Mode of action of linear amphipathic alpha-helical antimicrobial peptides. Biopolymers 47, 451–463 10333737 10.1002/(SICI)1097-0282(1998)47:6<451::AID-BIP4>3.0.CO;2-F

[B22] Robinson M. W. Alvarado R. To J. Hutchinson A. T. Dowdell S. N. Lund M. (2012). A helminth cathelicidin-like protein suppresses antigen processing and presentation in macrophages via inhibition of lysosomal vATPase. FASEB J. 26, 4614–4627 10.1096/fj.12-213876 22872675

[B23] Robinson M. W. Dalton J. P. Donnelly S. (2008a). Helminth pathogen cathepsin proteases: it's a family affair. Trends Biochem. Sci. 33, 601–608 10.1016/j.tibs.2008.09.001 18848453

[B24] Robinson M. W. Tort J. F. Wong E. Donnelly S. M. Lowther J. Xu W. (2008b). Proteomics and phylogenetic analysis of the cathepsin L protease family of the helminth pathogen, *Fasciola hepatica*: expansion of a repertoire of virulence-associated factors. Mol. Cell. Proteomics 7, 1111–1123 10.1074/mcp.M700560-MCP200 18296439

[B25] Robinson M. W. Dalton J. P. O'Brien B. A. Donnelly S. (2013). *Fasciola hepatica*: the therapeutic potential of a worm secretome. Int. J. Parasitol. 43, 283–291 23220126 10.1016/j.ijpara.2012.11.004

[B26] Robinson M. W. Donnelly S. Hutchinson A. T. To J. Taylor N. L. Norton R. S. (2011). A family of helminth molecules that modulate innate cell responses via molecular mimicry of host antimicrobial peptides. PLoS Pathog. 7:e1002042 10.1371/journal.ppat.1002042 21589904 PMC3093369

[B27] Robinson M. W. Menon R. Donnelly S. M. Dalton J. P. Ranganathan S. (2009). An integrated transcriptomic and proteomic analysis of the secretome of the helminth pathogen, *Fasciola hepatica*: proteins associated with invasion and infection of the mammalian host. Mol. Cell. Proteomics 8, 1891–1907 10.1074/mcp.M900045-MCP200 19443417 PMC2722771

[B28] Schroder K. Sweet M. J. Hume D. A. (2006). Signal integration between IFNgamma and TLR signalling pathways in macrophages. Immunobiology 211, 511–524 10.1016/j.imbio.2006.05.007 16920490

[B29] Sood R. Kinnunen P. K. (2008). Cholesterol, lanosterol, and ergosterol attenuate the membrane association of LL-37(W27F) and temporin L. Biochim. Biophys. Acta 1778, 1460–1466 10.1016/j.bbamem.2008.02.014 18358828

[B30] Thivierge K. Cotton S. Schaefer D. A. Riggs M. W. To J. Lund M. E. (2013). Cathelicidin-like helminth defence molecules (HDMs): absence of cytotoxic, anti-microbial and anti-protozoan activities imply a specific adaptation to immune modulation. PLoS Negl. Trop. Dis. 7:e2307 10.1371/journal.pntd.0002307 23875042 PMC3708846

